# Is Insanity Increasing in Scotland?

**Published:** 1895-03-30

**Authors:** 


An Extra Nursing Supplement.

Vol. XVII.—No. 444.] [March 30, 1895.

### Is Insanity Increasing in Scotland?

No one who has well considered the facts will deny that the place of Scotland is very near the apex of the structure of modern civilisation. In general education, in strenuousness, and in practical effectiveness her people are, perhaps, second to none. Can Scotland stand the strain which the civilisation of the period is putting upon all peoples? Could she, if circumstances were to demand it, stand a still severer strain? How does she compare with England in this respect, and how with other European countries? If, as seems probable, Scotland has stood the strain of civilisation better than some other European countries, what is the reason? Are we to look to the situation of the country, or to the characteristics of the people, to their habits in food, drink, and work, to their mental endowments and their religion, to any or to all of these? It is clear that an inquiry into the mental condition of Scotland, as to the influence upon her of the strenuous civilisation of our times, opens up many questions of the profoundest interest to those whose business it is, or whose curiosity prompts them, to ask seriously what is the ultimate goal to which modern civilisation is tending?

No other single test supplies us with so convincing a criterion of the power of a people to bear strain, or of the severity of the strain, as the sanity test. Is insanity on the increase in Scotland? It has been alleged, and persistently alleged, that it is. If it be, then either the strain of civilisation is becoming unendurable or the Scottish people are deteriorating in quality and physique. The question may seem to be one which concerns Scotland exclusively. But it is not so by any means. For it would not be difficult to maintain this thesis, that if Scotland has reached the limit of her capacity for bearing the strain of modern civilisation, other European nations have reached, or will soon reach, the limit of their capacity. And what is it which lies before Europe and the world in that event?

An authoritative answer as to the mental condition of the Scottish people in respect of sanity is of importance, not to Scotland only, but to England, Europe, and the civilised world. It is not improbable that the compilers of the "Supplement" to the Thirty-sixth Annual Report of the Scottish Lunacy Board, which has just been issued as a Blue Book, have taken this view of their work and its responsibilities. So far as has been possible, they have studied and utilised the facts and statistics of a period of nearly forty years, that is to say, a period extending from 1857 to 1894. They have had in their minds the oft-repeated statement that lunacy has been markedly on the increase in Scotland during the last three or four decades; and they have left nothing undone which industry and ingenuity could suggest in order that they might give an authoritative pronouncement on the subject.

The members of the Lunacy Board report "that the facts and figures, so far as they have been already collected and studied, afford no ground for a belief that insanity is to-day more prevalent in Scotland than it was when we entered upon our functions over thirty-six years ago." Those who are acquainted with the members of the Scottish Lunacy Board would be very well content to accept their conclusions without being at the labour to wade through the accumulated facts and arguments. But a glance at the methods by which they have arrived at their conclusions, and at the explanation they give of the apparent increase which has been so mistakenly accepted as proof of a real increase in lunacy, will well repay a little trouble.

The medical experts of the Board, who are mainly responsible for its conclusions, have approached the task of investigation from three different and independent standpoints. Sir Arthur Mitchell has taken a typical town parish, and studied its lunacy statistics for a period of ten years. Those statistics show beyond question that there was a decidedly larger number of registered lunatics in proportion to population at the end of the ten years than at the beginning. Apparently, then, lunacy had increased in that parish. Not at all, says Sir Arthur Mitchell. There had been no increased production of lunatics, but only a diminution in their death-rate, and in the proportion of returns of lunatics to the general population. In other words, a relatively larger proportion of Scottish lunatics are now sent to asylums than formerly; they are better taken care of in asylums, and so live longer; and they are not discharged from asylums to be returned to the general population. Thus there is an increased "accumulation" of lunatics in asylums; but no increased "production" of fresh lunatics.

Dr. Sibbald, as his contribution to the inquiry, has studied the lunacy statistics of the whole of Scotland from 1857 to 1894. Space does not permit us to give even an outline of the many and important facts which he has accumulated. It is important, however, to mention that, owing to the altered distribution of pauperism and pauper lunacy in Scotland, some districts show a marked increase in the number of registered lunatics, whilst other districts, in every respect similar, show an even more marked diminution. It is impossible that lunacy should have both increased and diminished. Dr. Sibbald concludes that the "statistics afford no evidence that mental unsoundness is to-day more prevalent in Scotland than it was in 1858."

Mr. Spence, the third of the medical experts, devoted his investigations to the "statistics relating to registered private patients." His conclusions agree unreservedly with those of the other medical experts.

From these, and from many other considerations put forward in the "Blue Book," it may be taken as proved that there is no increase at all of lunacy in Scotland. It is thus demonstrated that Scotland has stood the severe strain of modern civilisation without impairment of the mental powers of her population. It is probable that she could stand, if necessary, a still severer strain. If now a similar and equally trustworthy investigation were carried out in England and Ireland, and also in other European countries, information of the highest scientific and practical value would be accumulated, comparisons could be made, and conclusions arrived at which would be of the very first importance to the general progress of our whole European civilisation.

